# NLRP3 Inflammasome as Potential Predictor of Non-Responsiveness to Immunosuppressive Treatment in Lupus Nephritis

**DOI:** 10.3390/ijms27010043

**Published:** 2025-12-19

**Authors:** Camila Barbosa Lyra de Oliveira, Werbson Lima Guaraná, Gisele Vajgel, Braziliano Miguel da Silva Júnior, Camilla Albertina Dantas Lima, Stanley de Almeida Araújo, Fabrício Oliveira Souto, Denise Maria do Nascimento Costa, Lucila Maria Valente, Paula Sandrin-Garcia

**Affiliations:** 1 Division of Nephrology, Clinical Hospital, Federal University of Pernambuco, Recife 50670-901, Pernambuco, Brazil; camilabarbosalyra@hotmail.com (C.B.L.d.O.); gisele.vajgel@ebserh.gov.br (G.V.); lucilamvalente@gmail.com (L.M.V.); 2 Programa de Pós-Graduação em Biologia Aplicada à Saúde (PPGBAS), Federal University of Pernambuco, Recife 50670-901, Pernambuco, Brazil; braziliano.junior@ufpe.br; 3 Institute Keizo Asami (iLIKA), Federal University of Pernambuco, Recife 50670-901, Pernambuco, Brazil; werbson.guarana@ufpe.br (W.L.G.); camilla.lima@ufpe.br (C.A.D.L.); 4 Department of Oceanography, Federal University of Pernambuco, Recife 50670-901, Pernambuco, Brazil; 5 Nephropathology Institute, Belo Horizonte 30150-281, Minas Gerais, Brazil; stanleyaa@gmail.com; 6 Agreste Science Center, Federal University of Pernambuco, Recife 50670-901, Pernambuco, Brazil; fabricio.souto@ufpe.br; 7 Department of Genetics, Federal University of Pernambuco, Recife 50670-901, Pernambuco, Brazil

**Keywords:** systemic lupus erythematosus, lupus nephritis, NLRP3 inflammasome, disease activity, biomarker, prognosis

## Abstract

Lupus nephritis (LN) can affect up to 60% of patients with systemic lupus erythematosus (SLE). The NLRP3 inflammasome has been implicated in the pathogenesis of LN. This study aimed to evaluate the role of the NLRP3 inflammasome as a predictor of response to immunosuppressive treatment in patients with active LN. A prospective cohort study was conducted with 20 adult patients with active LN, classes III, IV, and V, from January 2021 to September 2023. Patients were followed up at biopsy (T0) and 6 months (T6) and 12 months (T12) after treatment and classified according to the primary efficacy renal response (PERR) at 12 months. Gene expression of *NLRP3*, *CARD8*, *CASP1*, *IL1B*, and *IL18* was evaluated by RT-qPCR in PBMCs. Immunohistochemistry (IHC) for NLRP3 was performed on kidney tissue. The concentration of cytokine IL-1β was measured using the BD™ Cytometric Bead Array (CBA). The mean age was 31.9 ± 8.3 years, with 19 females and 1 male. After 12 months, 65% of patients achieved PERR. The IHC intensity in inflammatory cells was higher in patients with no PERR (*p =* 0.0426). In the no-PERR group, the gene expression of *IL1B* showed a significant increase at T6 (FC = 2.22: *p* = 0.0037) and T12 (FC = 2.91; *p* = 0.0001) compared with T0. Relative expression of *IL1B* was higher in no-PERR patients at T12 compared to the PERR group (*p* = 0.0477). The no-PERR group also had higher serum IL-1β levels compared to the PERR group at 12 months (2.9 ± 0.5 vs. 2.5 ± 0.7, *p* = 0.0164). In conclusion, our study evidenced an increase in *IL1B* expression and IL-1β levels over the 12 months of treatment in no-PERR patients, suggesting a potential biomarker of disease activity. Furthermore, a strong NLRP3 IHC staining score was associated with a higher likelihood of no PERR, highlighting the potential of the NLRP3 inflammasome as a predictor of worse clinical outcomes.

## 1. Introduction

Lupus nephritis (LN) is a common and severe complication of systemic lupus erythematosus (SLE), affecting up to 60% of patients and leading to significant morbidity and mortality [1,2,3,4]. Despite advances in the knowledge of LN pathogenesis, the prognosis of LN patients remains poor, with a high risk of end-stage kidney disease and death [1]. LN is characterized by complex immune deposition and kidney inflammation, leading to kidney damage. The inflammasome, a multiprotein complex that plays a crucial role in the innate immune response, has been implicated in the pathogenesis of LN [5,6,7,8,9]. Among the various inflammasome complexes, NLRP3 has emerged as a key player in LN, with its activation contributing to the development of proteinuria, podocyte injury, tubular damage, and renal fibrosis [10,11,12,13].

The NLRP3 inflammasome comprises a pattern recognition receptor (PRR), an adaptor protein, and an effector protein. The nucleotide-binding and oligomerization domain (NOD)-like receptor family pyrin domain containing 3 (NLRP3) is an intracellular PRR activated in response to pathogen-associated molecular patterns (PAMPs) and/or damage-associated molecular patterns (DAMPs). When activated, NLRP3 recruits the adaptor protein ASC (apoptosis-associated speck-like protein containing a CARD) and pro-caspase, comprising the inflammasome complex [14].

This multiprotein complex activates caspase-1, an enzyme responsible for the cleavage of the pro-inflammatory cytokines pro-IL-1β and pro-IL18 into their active forms (IL-1β and IL18) [15,16]. Caspase-1 can also activate gasdermin D, a protein responsible for forming pores in the cell membrane and disrupting the cell membrane integrity. This process results in cell lysis and the release of cytoplasmic contents, including inflammatory cytokines, a process known as pyroptosis [17]. This mechanism is responsible for amplifying the inflammatory response, recruiting and activating additional immune cells, and causing further inflammation and greater damage [18].

Previous studies have established an association between *NLRP3* gain-of-function polymorphisms and increased susceptibility to developing SLE and LN [19,20,21,22]. Functional studies have demonstrated a correlation between elevated levels of the NLRP3 inflammasome and tissue damage in animal and human models [7,9,23]. Additionally, several studies have been conducted using NLRP3 inflammasome inhibitors in animal models to evaluate the effect of blocking this pathway on kidney tissue. These studies revealed a reduction in inflammasome activation, decreased expression of *IL1B*, *IL18*, and *CASP1*, a reduction in glomerular inflammation, and a reduction in proteinuria following the use of inflammasome inhibitors [24,25,26,27,28].

Given the increasing evidence, the NLRP3 inflammasome has been demonstrated to play an essential role in LN pathophysiology. Once activated, the NLRP3 inflammasome enhances inflammatory response, leading to significant kidney damage in both human and animal models. Despite these findings, the expression of the NLRP3 inflammasome during immunosuppressive treatment remains poorly established. This study aimed to evaluate the contribution of the NLRP3 inflammasome as a predictor of treatment response in active LN patients.

## 2. Results

### 2.1. Clinical and Laboratory Characteristics

**Figure 1:**
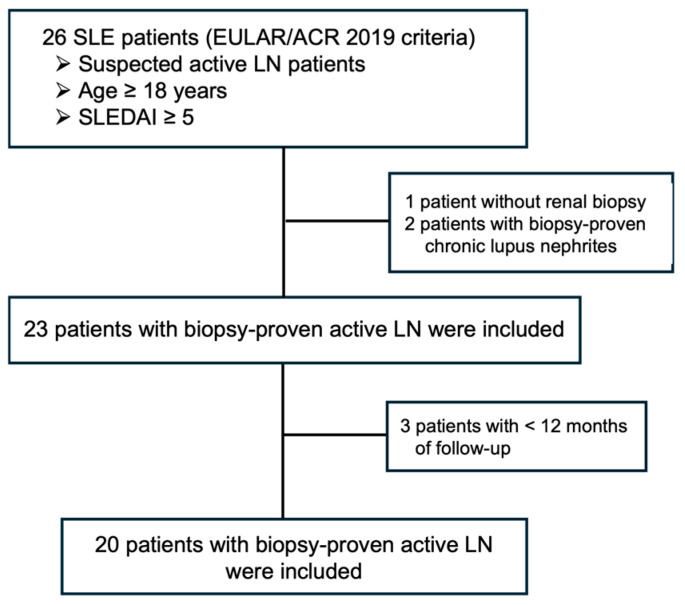
Flowchart of patient enrollment. SLE, systemic lupus erythematosus; EULAR/ACR, European Alliance of Associations for Rheumatology/American College of Rheumatology [29]; LN, lupus nephritis; SLEDAI, systemic lupus erythematosus disease activity index [30].

Twenty-six patients were selected for evaluation during the period with suspected active lupus nephritis. Three patients failed to meet the inclusion criteria: one patient did not undergo kidney biopsy due to the severity of hematological manifestations (thrombocytopenia and anemia) and two patients did not have active lupus nephritis lesions in their kidney biopsy. Three patients were excluded due to having a follow-up period of less than 12 months (Figure 1). A total of 20 active LN patients were followed up for 12 months of immunosuppressive treatment.

The mean age was 31.9 ± 8.3 years, with 19 females and 1 male. Laboratory evaluation showed a median serum creatinine (sCr) of 0.8mg/dL (0.7–1.3), a median estimated glomerular filtration rate (eGFR) of 103 mL/min/1.73m^2^ (54–119), and a median 24-h urine protein level of 3.3g (1.7–4.9). The mean systemic lupus erythematosus disease activity index (SLEDAI) score was 14.6 ± 4.4, and 95% of patients had complement C3 consumption (mean C3 57.2 ± 22.6 mg/dL). Class IV was the most frequent histological pattern, occurring in 11 (55%) patients (7 patients—LN class IV; 4 patients—LN IV + V). LN class III was observed in 5 (25%) patients (3 patients—LN class III; 2 patients—LN III + V), while pure class V was present in 4 (20%) patients. Twelve (60%) patients received intravenous cyclophosphamide (CYC) as initial treatment, and eight (40%) patients received MMF. All patients received mycophenolate mofetil (MMF) for maintenance treatment.

After 12 months of immunosuppressive treatment, 13 (65%) patients achieved primary efficacy renal response (PERR). In this way, the patients were divided into two groups according to treatment response (PERR and no PERR). Clinical, laboratory, and treatment characteristics were compared between the groups at baseline (Table 1). Patients with no PERR had a longer duration of LN compared to the PERR group (*p* = 0.0115).

### 2.2. Histopathological Findings

Histopathological characteristics, the International Society of Nephrology/Renal Pathology Society (ISN/RPS) 2004 classification [31,32], activity index (AI), chronicity Index (CI) [33], and immunohistochemistry (IHC) data from baseline kidney biopsies were also compared based on treatment response (Table 2). The most common LN classification was class IV ± V, which was present in the majority of patients from both groups. LN class V, whether pure or in association with class III or IV, was more frequent in the no-PERR group (85.7%) compared to the PERR group (30.8%), although this difference was not statistically significant (*p* = 0.573). Cellular and fibrocellular crescents were present in nearly 65% of patients in both groups (PERR and no PERR) (*p* = 0.6514). The groups had a similar distribution of AI, CI, and specific histopathological features, indicating similar active disease and chronic damage at baseline.

IHC staining of NLRP3 from baseline kidney biopsies is listed in Table 3. NLRP3 staining showed significantly different intensities in inflammatory cells between PERR and no-PERR patients (*p* = 0.0426). Most PERR patients had weak positive (+1) (53.8%) or negative (30.8%) NLRP3 staining in inflammatory cells. In contrast, the majority of the no-PERR group had strong positive (+2) NLRP3 staining in inflammatory cells, with a significant difference compared to the PEER group (57.2% vs. 15.4%, *p* = 0.0210). Patients with strong positive (+2) NLRP3 staining in the inflammatory cells had a 3 times higher risk of not achieving PERR compared to patients with weak positive (+1) NLRP3 staining (RR 3.0–CI 1.4–10.3; *p* = 0.0210).

**Figure 2:**
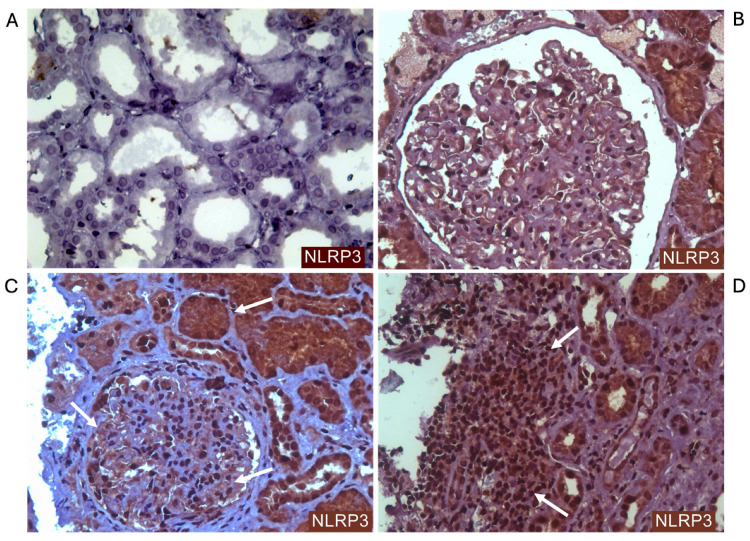
Immunohistochemistry (IHC) staining of NLRP3 in renal biopsy from patients with active lupus nephritis. The brown coloration represents areas where NLRP3 is present, suggesting active inflammasome involvement. (**A**) Renal cortex with multiple tubules and normal tubular epithelial cells. Negative control. Magnification 400×. (**B**) Glomerulus with positive NLRP3 staining. Magnification 400×. (**C**) Positive NLRP3 staining in mesangial and glomerular capillary areas (arrows). The surrounding tubules also exhibit NLRP3 staining. Magnification 400×. (**D**) Interstitial infiltration of inflammatory cells and tubular cells with positive NLRP3 staining (arrows). Magnification 400×.

All patients had positive NLRP3 staining in tubular cells, and most patients in both groups had strong staining intensity. There were no statistical differences between groups in NLRP3 staining intensity in tubular cells. Compared with the PERR group, patients in the no-PERR group demonstrated a higher frequency of podocytes with strong (+2) NLRP3 staining (28.6% vs. 7.7%; *p* = 0.2701), although this difference was not statistically significant. Figure 2 shows IHC staining of NLRP3 in kidney tissue from active LN patients.

### 2.3. Clinical and Laboratory Characteristics During Follow-Up

The laboratory data were compared across the groups at 6 months and 12 months after kidney biopsy and immunosuppressive treatment (Table 4). After 6 months, the no-PERR group had lower eGFR (*p* = 0.0165), lower serum albumin (*p* = 0.0218), and higher levels of proteinuria (*p* = 0.0037) compared to the PERR group. After 12 months, patients with no PERR had higher SLEDAI scores (*p* = 0.004) and higher levels of proteinuria compared to patients with PERR. Most patients in both groups received MMF and corticosteroids in similar doses after 6 months of treatment.

### 2.4. Inflammasome Gene Expression Analysis

**Figure 3:**
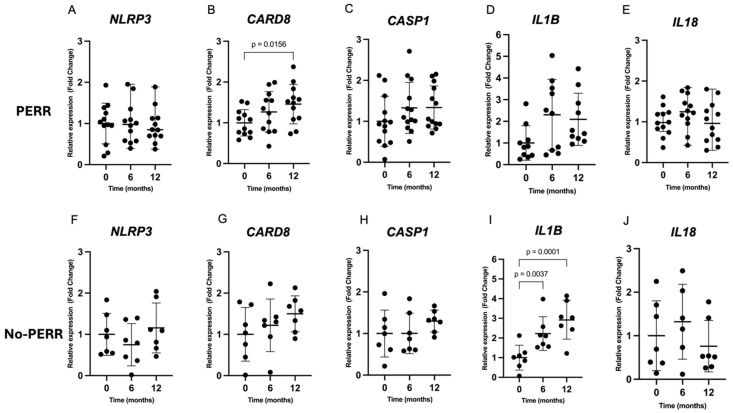
Gene expression profile of NLRP3 inflammasome genes and downstream cytokines in LN patients according to treatment response (PERR or no PERR) at baseline and 6 months and 12 months after kidney biopsy with active lupus nephritis and immunosuppressive treatment. PERR group: (**A**) *NLRP3*; (**B**) *CARD8*; (**C**) *CASP1*; (**D**) *IL1B*; (**E**) *IL18*. No-PERR group: (**F**) *NLRP3*; (**G**) *CARD8*; (**H**) *CASP1*; (**I**) *IL1B*; (**J**) *IL18*. PERR: primary efficacy renal response.

Relative gene expression over 12 months in patients with PERR and no PERR is shown in Figure 3. The relative gene expression of *NLRP3*, *CASP1*, *IL1B*, and *IL18* did not demonstrate significant changes over 12 months of immunosuppressive treatment in the PERR group. Despite this, *CARD8* expression significantly increased over 12 months in the PERR group (FC = 1.45; *p* = 0.0156) (Figure 3C).

In the no-PERR group, the gene expression of *IL1B* showed a significant increase after 6 months (FC = 2.22; *p* = 0.0037) and 12 months (FC = 2.91; *p* = 0.0001) of treatment compared with the baseline (T0) (Figure 3I). A repeated-measures analysis of variance of *IL1B* expression revealed a statistically significant difference between 0, 6, and 12 months (*p* = 0.0002). The gene expression of *NLRP3*, *CARD8*, *CASP1*, and *IL18* did not significantly differ over the analyzed period. The comparison of relative gene expression between the PERR and no-PERR groups at baseline, 6 months, and 12 months was evaluated. *NLRP3*, *CARD8*, *CASP1*, and *IL18* expression did not differ between the groups over the 12 months of follow-up. However, after 12 months of treatment, *IL1B* expression was significantly higher in patients with no PERR compared with those with PERR (FC 1.57; *p =* 0.0477).

**Figure 4:**
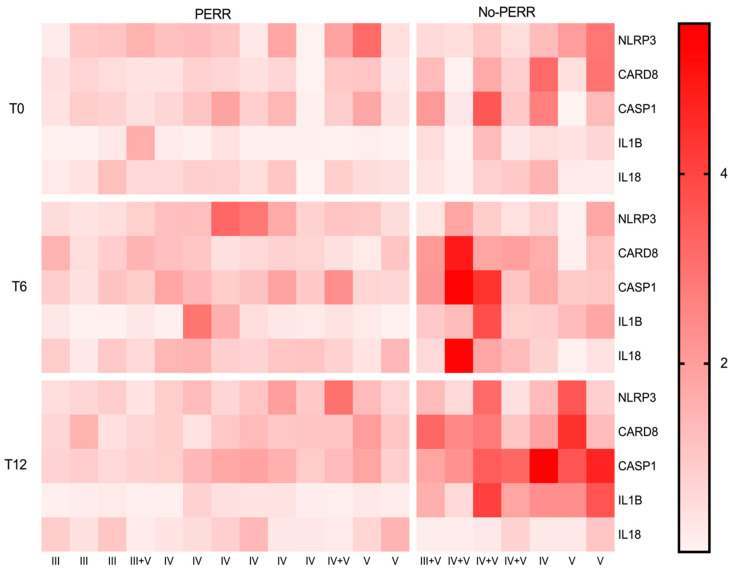
Heatmap representing expression of NLRP3 inflammasome genes and downstream cytokines in lupus nephritis (LN) patients according to treatment response (PERR and no PERR) at 3 time points after kidney biopsy (T0, T6, and T12) and immunosuppressive treatment. PERR: primary efficacy renal response; T0: baseline; T6: 6 months; T12: 12 months; III: NL class III; IV: LN class IV; V: LN class V.

Figure 4 presents a heatmap representing the gene expression of NLRP3 inflammasome-related genes (*NLRP3*, *CARD8*, *CASP1*) and cytokine genes (*IL1B*, *IL18*) over 12 months of treatment. The data were divided into two groups according to treatment response (PERR and no PERR) at three time points after kidney biopsy and initial immunosuppressive therapy (T0: baseline; T6: 6 months; T12: 12 months). Lighter shades of red represent lower expression levels, and darker shades correspond to higher gene expression levels. At baseline, gene expression levels were generally low for both the PERR and no-PERR groups. Across the time points, the PERR group maintained stable gene expression for most genes. In contrast, the no-PERR group showed an increase in color intensity in months 6 and 12, representing a higher expression of inflammasome genes.

### 2.5. Serum Levels of IL-1β

**Figure 5:**
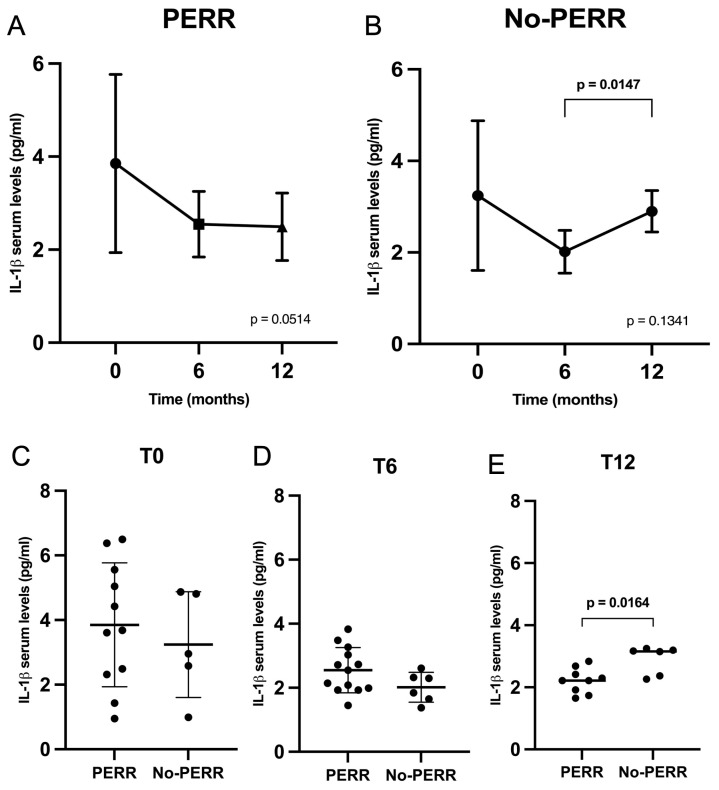
Serum levels of IL-1β in lupus nephritis patients. (**A**) Serum levels of IL-1β in PERR group over 12 months. (**B**) Serum levels of IL-1β in no-PERR group over 12 months. Comparative analyses of serum levels of IL-1β in PERR and no-PERR groups at baseline (**C**), 6 months (**D**), and 12 months (**E**). PERR, primary efficacy renal response.

The serum levels of IL-1β in the PERR and no-PERR groups over 12 months are represented in Figure 5A,B. The PERR group exhibited a decrease in serum IL-1β within the first 6 months (3.9 ± 1.9 vs. 2.6 ± 0.7—*p* = 0.1255) and remained stable until 12 months (2.6 ± 0.7 vs. 2.5 ± 0.7—*p* = 0.9483). In the no-PERR group, IL-1β levels decreased in the first 6 months (3.2 ± 1.6 vs. 2.0 ± 0.5—*p* = 0.3076) but subsequently increased until 12 months (2.0 ± 0.5 vs. 2.9 ± 0.5—*p* = 0.0147).

The comparative analyses of serum levels of IL-1β in the PERR and no-PERR groups at baseline, 6 months, and 12 months are demonstrated in Figure 5C–E. No significant differences in IL-1β serum levels were observed at baseline and 6 months. However, LN patients with no PERR had higher serum IL-1β levels compared to patients with PERR at 12 months (2.5 ± 0.7 vs. 2.9 ± 0.5—*p* = 0.0164).

### 2.6. Correlation Analysis

The correlations between clinical, laboratory, and histopathological parameters at baseline, 6 months, and 12 months after kidney biopsy and initial immunosuppressive treatment were tested. Evaluating all patients at baseline (PERR and no-PERR groups), *NLRP3* expression had a negative correlation with corticosteroid doses (r = 0.5344, *p* = 0.0184), and *CARD8* expression was positively correlated with SLEDAI (r = 0.5084, *p* = 0.0262). *IL18* expression had a positive correlation with fibrocellular/cellular crescents (r = 0.6257, *p* = 0.0042) and with the CI (r = 0.5834, *p* = 0.0087). In month 6, *IL18* expression correlated positively with serum creatinine (r = 0.5592, *p* = 0.0128) and with serum IL-1β (r = 0.4760, *p* = 0.0459).

**Figure 6:**
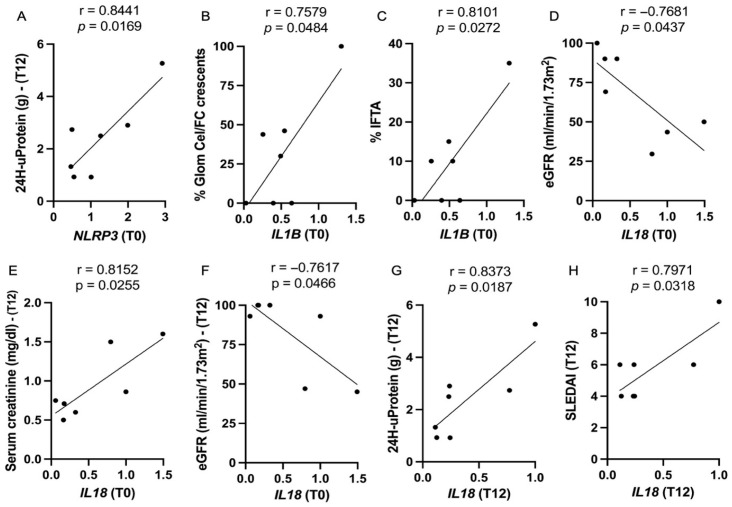
Correlation analysis between gene expression of *NLRP3*, *CARD8*, *CASP1*, *IL1B*, and *IL18* and clinical and histopathological parameters in lupus nephritis patients with no PERR. Only statistically significant results are shown. (**A**) *NLRP3* (baseline) and 24 h uProtein (12 months); (**B**) *IL1B* (baseline) and % glomeruli with cellular and fibrocellular crescents; (**C**) *IL18* (baseline) and %IFTA; (**D**) *IL18* (baseline) and eGFR (baseline); (**E**) *IL18* (baseline) and serum creatinine (12 months); (**F**) IL18 (baseline) and eGFR (12 months); (**G**) *IL18* (12 months) and SLEDAI (12 months); (**H**) *IL18* (12 months) and 24 h uProtein (12 months). 24 h uProtein: 24-h urine protein; Glom, glomeruli; Cel/FC: cellular and fibrocellular; IFTA, interstitial fibrosis and tubular atrophy; eGFR, estimated glomerular filtration rate; SLEDAI, systemic lupus erythematosus disease activity index; T0, baseline; T12, 12 months.

Evaluating the group with no PERR, baseline *NLRP3* expression exhibited a strong positive correlation with 12-month 24 h proteinuria (r = 0.8441, *p* = 0.0164) (Figure 6A). *IL1B* expression was positively correlated with cellular/fibrocellular crescents (r = 0.7579, *p* = 0.0484) and with the extent of interstitial fibrosis and tubular atrophy (r = 0.8101, *p* = 0.0272), lesions linked to severe histopathological damage (Figure 6B,C).

The analysis of *IL18* expression at baseline revealed a strong negative correlation with initial eGFR (r = −0.7681; *p* = 0.0437) and with 12-month eGFR (r = 0.7617, *p* = 0.0466), indicating that higher *IL18* expression was correlated with lower kidney function at baseline and after 12 months of immunosuppressive treatment (Figure 6D–F). In addition, *IL18* expression at 12 months had a strong positive correlation with SLEDAI (r = 0.7971, *p* = 0.0318) and 24 h proteinuria (r = 0.8373, *p* = 0.0187) (Figure 6G,H).

## 3. Discussion

Several studies have demonstrated an upregulation of the NLRP3 inflammasome in the kidney tissue of patients with LN, correlating with tissue damage and disease activity [7,9,23]. Despite these findings, NLRP3 inflammasome expression over the course of immunosuppressive treatment and its potential in predicting the prognosis of LN are not well-established. In our study, we observed a progressive increase in *IL1B* expression and serum IL-1β levels in the no-PERR group, suggesting a persistent inflammatory state. We also demonstrated that strong NLRP3 staining intensity in renal tissue was associated with no PERR after 12 months of therapy, indicating its potential prognostic value.

At baseline, both groups had similar clinical and laboratory characteristics, except for a longer duration of LN in the no-PERR group. Although this could suggest greater chronicity, the CI and specific chronic lesions did not differ between groups. The trend toward higher baseline creatinine in the no-PERR group (1.0 vs. 0.7; *p =* 0.0664) may reflect more severe disease, but proteinuria, SLEDAI, and activity index (AI) were comparable, indicating similar baseline disease severity.

NLRP3 immunostaining was positive in the tubular cells of all patients, with strong intensity in over 70% of patients, and this was also observed in podocytes and inflammatory cells in most LN cases. These findings are consistent with previous studies demonstrating NLRP3 inflammasome activation in both immune and intrinsic kidney cells, with associated *IL-1β* and *IL-18* expression [9,28,34,35,36]. Importantly, patients with no PERR exhibited significantly stronger NLRP3 staining in inflammatory cells, which was associated with a threefold higher risk of non-responsiveness. Although strong podocyte staining was more frequent in the no-PERR group, it did not reach statistical significance. Baseline *NLRP3* and *IL18* expression levels also correlated with key laboratory markers of prognosis, supporting the relevance of NLRP3 activation intensity as a prognostic marker in active LN.

Studies using animal models have demonstrated the contribution of NLRP3 inflammasome activation to kidney inflammation, tubulointerstitial fibrosis, glomerulosclerosis, and progression of chronic kidney disease (CKD) [12,34]. NLRP3 inflammasome activation in immune and intrinsic kidney cells induces IL-1β and IL-18 release, promotes macrophage recruitment, amplifies renal inflammation, and triggers caspase-1-mediated pyroptosis via gasdermin-D cleavage [35,36]. In podocytes, IL-1β has been shown to downregulate nephrin expression, thereby interfering with podocyte structural integrity, promoting podocyte injury, and contributing to the development of proteinuria [37,38,39].

Activation of the NLRP3 inflammasome in tubular epithelial cells induces the release of pro-inflammatory cytokines, particularly IL-1β, which promotes transforming growth factor-β1-mediated fibrogenesis (TGF-β1), extracellular matrix deposition, and tubulointerstitial fibrosis [40,41,42]. These mechanisms contribute to interstitial fibrosis, tubular atrophy, persistent proteinuria, and progressive loss of kidney function [43]. This biological pathway supports our finding that stronger NLRP3 staining is associated with a lower probability of renal response, likely reflecting more intense inflammation, greater tissue damage, and worse clinical outcomes.

In the no-PERR group, higher baseline *IL1B* expression correlated with greater histopathological severity, including crescents, interstitial fibrosis, and tubular atrophy, while elevated *IL18* expression was associated with worse baseline eGFR [43,44,45,46]. These findings indicate that increased NLRP3 inflammasome activation is linked to both acute and chronic kidney injury and impaired renal function, consistent with previous studies [12,47,48]. At 12 months, the no-PERR group exhibited higher *IL1B* gene expression, increased serum IL-1β levels, greater proteinuria, and higher SLEDAI scores compared to the PERR group. Despite overall low SLEDAI values, these differences indicate a more persistent inflammatory state in the no-PERR group, likely driven by sustained NLRP3 inflammasome activation and associated with poorer renal outcomes.

It is noteworthy that while IL-1β is a general inflammatory cytokine, its specific upregulation pattern—recorded over the duration of immunosuppressive treatment and correlating with renal NLRP3 staining intensity—supports the notion that the NLRP3/IL-1β axis may play a distinct role in mediating treatment resistance in LN, rather than merely reflecting a non-specific systemic inflammatory state. This strengthens the pathway’s potential as a specific therapeutic target and biomarker in lupus nephritis.

Given the increasing relevance of the NLRP3 inflammasome in the pathogenesis of LN, studies evaluating the effect of NLRP3 inflammasome blockers in kidney tissue have increased over the years. In an animal model, treatment with the selective NLRP3 inhibitor MCC950 suppressed NLRP3, caspase-1, and IL-1β activation in podocytes, resulting in reduced proteinuria and significant improvement in histological damage and foot process effacement compared to controls [38,49]. Other studies have shown that different drugs reduce proteinuria and attenuate renal damage, reinforcing the direct pathogenic role of NLRP3 inflammasome activation in kidney inflammation and podocyte injury [50,51,52].

Another relevant finding was the progressive increase in *CARD8* expression in the PERR group, accompanied by lower IL-1β levels and reduced proteinuria. This observation could be attributed to the inhibitory effects of caspase recruitment domain-containing protein 8 (CARD8) on the NLRP3 inflammasome [53]. CARD8 has been characterized as an inflammasome sensor associated with anti-inflammatory and anti-apoptotic activities. CARD8 can bind and inhibit caspase-1, resulting in negative regulation of IL-1β activation. CARD8 also interacts with a nuclear factor kappa B (NF-kB) modulator and inhibits NF-κB activity, a key mediator of the priming signal required for the NLRP3 inflammasome. In addition, CARD8 can directly interact with NLRP3, preventing its binding to the adaptor ASC and inhibiting the activation of the NLRP3 inflammasome [54,55].

Gain-of-function mutations in *NLRP3* and loss-of-function mutations in *CARD8* disrupt their inhibitory interaction, leading to enhanced inflammasome activity and increased IL-1β secretion [55,56]. These mutations may promote a pro-inflammatory phenotype by sustaining NLRP3 inflammasome activation, leading to increased *IL1B* expression, higher IL-1β levels, and greater proteinuria. Although initially described as an inhibitor, CARD8 can also assemble a functional inflammasome under specific conditions, suggesting a context-dependent dual role [57,58]. In our cohort, increased *CARD8* expression in responders may indicate a protective regulatory mechanism limiting inflammation.

This study has some limitations. Although prospectively followed up, the relatively small sample size may reduce the generalizability of the results. Additionally, the single-center design may limit the external validity, as the patient population may not fully reflect the heterogeneity of lupus nephritis in different clinical settings. Our gene expression analysis focused on a core set of NLRP3 inflammasome-related genes in PBMCs. It did not assess the contribution of alternative splice variants or a broader panel of inflammatory mediators, which might provide additional insights. Additionally, the observational and correlational design precludes causal inferences. Future studies with larger cohorts, longer follow-up, and multi-omics approaches are warranted to validate these preliminary findings and to explore the mechanistic links between NLRP3 activation and treatment resistance in lupus nephritis.

In conclusion, our findings suggest that stronger NLRP3 inflammasome staining may help identify patients at risk of no PERR and worse renal prognosis. Furthermore, our data suggest that IL-1β, in combination with clinical and laboratory parameters, could serve as a useful biomarker for monitoring disease activity and treatment response, contributing to more personalized management of LN.

## 4. Materials and Methods

### 4.1. Patients

We conducted a prospective cohort study of adult patients with SLE and kidney biopsy-proven active LN from January 2021 to September 2023. The inclusion criteria were age ≥ 18 years; diagnosis of SLE by EULAR/ACR 2019 [29]; SLEDAI ≥ 5; and kidney biopsy with active LN class III, IV, and/or V according to the ISN/RPS classification [31,32]. Exclusion criteria were inconclusive or inadequate biopsies (without glomeruli), follow-up time less than 12 months, patients with other autoimmune diseases, and patients with active infectious diseases.

Clinical characteristics, laboratory results, and treatment data were collected at the time of initial immunosuppressive treatment (T0), after 6 months (T6), and after 12 months (T12). Blood samples for gene expression and cytokine analysis were collected with the same frequency. Laboratory tests included serum creatinine, serum albumin, complement 3, complement component 4, urinalysis, urine protein/creatinine ratio, and 24-h urine protein.

Ethnicity was defined based on self-report and categorized as white and non-white (mixed or black). Disease activity was evaluated by SLEDAI [30]. The eGFR was calculated using the CKD Epidemiology Collaboration (CKD-EPI) equation [59]. Hypertension was defined as having a supine systolic blood pressure of ≥140 mmHg or a diastolic blood pressure of ≥90 mmHg in two consecutive measurements or treatment with antihypertensive drugs. SLE duration was defined as the interval from clinical and laboratory diagnosis to study enrollment [29]. LN duration was defined as the time from laboratory diagnosis (persistent proteinuria ≥ 500 mg/24 h or a protein-to-creatinine ratio ≥ 0.5, with or without urinary sediment abnormalities) to enrollment [60].

### 4.2. Histopathological Characteristics

The histological diagnoses were performed by an experienced kidney pathologist according to the ISN/RPS classification [31,32]. Glomerular and tubular lesions were evaluated using quantitative analysis. The AI and CI were based on the modified NIH activity and chronicity score system (AI range: 0–24; CI range: 0–12) [33]. Biopsy evaluation included immunofluorescence with staining for IgG, IgA, and IgM isotypes, kappa and lambda light chains, and complement components C3 and C1q.

### 4.3. Immunohistochemistry of Kidney Biopsy

Serial 4 mm histological sections were obtained from paraffin blocks and subsequently placed on salinized slides. The monoclonal antibodies IgE and NLRP3 were applied. The IHC technique involved the use of an amplified polymer according to the method recommended by the IHC Manual from the Brazilian Society of Pathology, and the slides were processed with diaminobenzidine. The background staining was performed with hematoxylin. Negative controls consisted of the replacement of primary antisera for rabbit or goat immunoglobulins of the same primary antibody class. IHC of NLRP3 was performed using primary antibodies against NLRP3 (AB_2746855; Thermofisher). NLRP3 staining intensity was graded on a scale of 0–2, according to the following assessment: no detectable staining (0), weak positive staining (1+), and strong positive staining (2+).

### 4.4. Treatment Protocols

The therapeutic regimen followed the standard hospital protocol, and treatment decisions were made exclusively by the attending physicians without any influence from the research team. Initial therapy consisted of high-dose glucocorticoids combined with either intravenous CYC or oral MMF, selected by the physician based on efficacy, safety, quality of life, and patient adherence. Glucocorticoid therapy included methylprednisolone pulses (0.25–0.5 g/day for 1–3 days, depending on disease severity), followed by oral prednisone (0.5–1.0 mg/kg/day).

CYC could be administered using a low-dose regimen (500 mg every 2 weeks for 3 months) or a high-dose regimen (0.5–1.0 g/m^2^ monthly for 6 months). MMF was initiated at 2–3 g/day, targeting a dose of 3 g/day. In cases of insufficient response after 3–6 months, physicians switched therapy from CYC to MMF or vice versa. Patients who responded to initial treatment received maintenance therapy with MMF (1–2 g/day) or azathioprine when MMF was contraindicated.

### 4.5. Definition of Treatment Response

After 12 months of immunosuppressive treatment, patients were classified according to their response, categorized as having PERR or no PERR. Kidney Disease Improving Global Outcomes (KDIGO) defines PERR as proteinuria ≤ 0.7 g/day and eGFR no worse than 20% below the pre-flare value or at least 60 mL/min/1.73 m^2^ without the use of rescue treatment for treatment failure. PERR has been associated with favorable long-term kidney outcomes in large cohorts, and its use is recommended by LN KDIGO guidelines [60,61,62,63].

### 4.6. Peripheral Blood Mononuclear Cell Isolation, RNA Extraction, and cDNA Synthesis

Peripheral Blood Mononuclear Cells (PBMCs) were isolated using Ficoll-Paque^®^ PLUS (GE Healthcare, Chicago, IL, USA) from 4 mL of peripheral blood collected in tubes containing EDTA. The RNA was extracted by TRIzol^®^ (Invitrogen, Carlsbad, CA, USA) according to the manufacturer’s instructions. The quantity and purity of total RNA were assessed separately. RNA concentration (quantification) was determined by spectrophotometry Nanodrop (ThermoFisher Scientific, Waltham, MA, USA) by measuring absorbance at 260 nm, and the samples were diluted to a working concentration of 100 ng/μL. Purity was evaluated based on the A260/A280 and A260/230 absorbance ratios, with only samples exhibiting values between 1.8 and 2.1 and greater than 2.0, respectively, being accepted [64]. Additionally, RNA integrity was assessed by electrophoresis on a 1% denaturing agarose gel containing formaldehyde to maintain RNA integrity. The samples were previously mixed with a denaturing loading buffer, heated at 65 °C for 10 min, and cooled on ice before loading. Electrophoresis was carried out at 5 V/cm for approximately 60 min in a submerged system containing 1X TBE buffer. After the run, the gel was visualized under ultraviolet light.

cDNA synthesis was performed with 500 ng of each RNA sample using the GoScript™ Reverse Transcription System (Promega, Madison, WI, USA), following the manufacturer’s instructions.

### 4.7. Gene Expression Analysis

The gene expression assays were performed with TaqMan^®^ fluorogenic probes to measure the target genes *NLRP3*, *CARD8*, *CASP1*, *IL1B*, and *IL18* (Thermo Fisher, Madison, WI, USA). “The TaqMan assay for NLRP3 (Hs00918082_m1) detects the canonical transcript variant encoding the full-length protein isoform. While multiple splice variants exist, this assay targets the predominant isoform associated with inflammasome activity”. The reference genes ribosomal protein P0 (*RPLP0*) and elongation factor 1-alpha 1 (*EF1A*) were used for data normalization. The experiments were performed on the ABI7500 real-time PCR system (Thermo Fisher, Madison, WI, USA).

Relative quantitative expressions were calculated following the fold change (FC) method suggested by Schmittgen and Livak for the assays: FC = 2^−ΔΔCq^ or ${\mathrm{FC}=2}^{-[(\mathrm{Cq}\;\mathrm{gene}\;\mathrm{of}\;\mathrm{interest}-{\mathrm{Cq}\;\mathrm{internal}\;\mathrm{control})}_{\mathrm{sample}\;A}-{(\mathrm{Cq}\;\mathrm{gene}\;\mathrm{of}\;\mathrm{interest}-\mathrm{Cq}\;\mathrm{internal}\;\mathrm{control})}_{\mathrm{sample}\;B}]}$ [65].

### 4.8. Serum IL-1β Quantification

Serum samples were collected at three time points (T0, T6, and T12) and stored at −80 °C until cytokine quantification was performed. The concentration of cytokine IL-1β was measured using the BD™ Cytometric Bead Array (CBA). The analyses were performed on the Accuri C6 flow cytometer (BD Biosciences, Franklin Lakes, NJ, USA).

### 4.9. Statistical Analysis

Normal distribution was tested using the Shapiro–Wilk test. Descriptive statistics were presented utilizing the mean ± standard deviation for parametric data and median and interquartile range for non-parametric data. Quantitative differences in paired gene expression samples were evaluated using one-way ANOVA or the Friedman test. Comparisons of continuous variables were conducted using the *t*-test or the Mann–Whitney test, depending on the normality of the distribution. Categorical variables were compared using Fisher’s exact test. The Pearson or Spearman correlation coefficient was used to evaluate the relationship between gene expression analysis and clinical, laboratory, and histopathological parameters. Statistical significance was defined as a *p*-value < 0.05. Statistical analysis was performed with GraphPad Prism (version 10.2.3).

## Figures and Tables

**Table 1:** Baseline characteristics of 20 patients with active lupus nephritis.

Characteristics	PERR N = 13	No PERR N = 7	*p*-Value
Age (years)	30 (24–39)	28 (26–43)	0.8618 *
Male/female	0/13	1/6	0.3500 ^‡^
Ethnicity, n (%)			
White	4 (30.8)	4 (57.1)	0.3563 ^‡^
Non-white	9 (69.2)	3 (42.9)	
Duration of SLE (months)	30 (16–146)	67 (33–114)	0.5486 ^¥^
Duration of LN, (months)	10.7 *±* 7.9	44.3 *±* 42.6	0.0115 *
Hypertension, n (%)	3 (23.1)	4 (57.1)	0.1736 ^‡^
Diabetes mellitus, n (%)	0 (0.0)	0 (0.0)	
BMI (kg/m^2^)	23 (22–24)	25 (20–27)	0.7569 ^¥^
Serum Cr (mg/dL)	0.7 (0.7–1.1)	1.0 (0.8–1.7)	0.0664 ^¥^
eGFR (mL/min/1.73 m^2^)	115 (76–121)	69 (44–103)	0.1191 ^¥^
Proteinuria (g/24 h)	3.6 ± 2.8	3.9 ± 2.6	0.7975 *
Serum albumin (g/dL)	2.8 ± 0.6	2.5 ± 0.8	0.4926 *
C3 (mg/dL)	50 ± 24	54 ± 21	0.6278 *
C4 (mg/dL)	7.5 (5.2–12.5)	8.0 (4.0–12.0)	0.9515 ^¥^
SLEDAI	15 ± 4	14 ± 5	0.8107 *
Previous immunosuppression, n (%)	4 (30.7)	5 (71.4)	0.1597 ^‡^
Use of ACEI/ARB, n (%)	10 (76.9)	7 (100)	0.5211 ^‡^
Use of hydroxychloroquine, n (%)	13 (100)	7 (100)	
Initial immunosuppressive treatment			
Corticosteroids dose (mg/kg/day)	0.7 ± 0.2	0.6 ± 0.2	0.4417 *
Methylprednisolone IV pulses	6 (46.2)	6 (85.7)	0.1577 *
Cyclophosphamide, n (%)	8 (61.6)	4 (57.1)	>0.999 ^‡^
MMF, n (%)	5 (38.4)	3 (42.9)	

Values expressed as median with range (25th–75th percentile) or mean ± standard deviation (SD). * *t*-test; ^¥^ Mann–Whitney test; ^‡^ Fisher’s exact test. PERR, primary efficacy renal response; SLE, systemic lupus erythematosus; LN, lupus nephritis; BMI, body mass index; Cr, creatinine; eGFR, estimated glomerular filtration rate; SLEDAI, systemic lupus erythematosus disease activity index; ACEI, angiotensin-converting enzyme inhibitors; ARB, angiotensin receptor blocker; MMF, mycophenolate mofetil. eGFR was calculated using the Chronic Kidney Disease Epidemiology Collaboration (CKD-EPI) equation. Hypertension was defined as systolic BP ≥ 140 or diastolic BP ≥ 90 mmHg, or treatment with antihypertensive drugs.

**Table 2:** Baseline histopathological characteristics of kidney biopsies from 20 patients with active lupus nephritis.

Histopathological Characteristics	PERR N = 13	No PERR N = 7	*p*-Value
Classification			
LN class III ± V, n (%)	4 (30.7)	1 (14.3)	0.6126 ^‡^
LN class IV ± V, n (%)	7 (53.9)	4 (57.1)	>0.999 ^‡^
LN class V, n (%)	2 (15.4)	2 (28.6)	0.2487 ^‡^
Activity index	9 ± 4	7 ± 3	0.5540 *
Chronicity index	2 (1–3)	3 (0–4)	0.7729 ^¥^
% Global glomerulosclerosis	0.0 (0.0–1.7)	0.0 (0.0–22)	0.3673 ^¥^
Endocapillary hypercellularity, n (%)	10 (76.9)	2 (28.6)	0.0623 ^‡^
% Endocapillary hypercellularity	22.2 (2.9–48.2)	0 (0.0–31.7)	0.0886 ^¥^
Subendothelial hyaline deposits, n (%)	5 (38.5)	2 (28.6)	>0.999 ^‡^
% Subendothelial hyaline deposits	0 (0–14.2)	0 (0–24)	0.8667 ^¥^
Cellular/fibrocellular crescents, n (%)	9 (69.2)	4 (57.1)	0.6514 ^‡^
% Cellular/fibrocellular crescents	16.7 (0.0–40.8)	14.3 (0.0–46.1)	0.4518 ^¥^
Interstitial inflammation, n (%)	7 (53.9)	3 (42.9)	>0.999 ^‡^
% Interstitial fibrosis/tubular atrophy	10 (0–10)	10 (0–50)	0.8920 ^¥^

Values expressed as median with range (25th–75th percentile) or mean ± standard deviation (SD). LN, lupus nephritis. * *t*-test; ^¥^ Mann–Whitney test; ^‡^ Fisher’s exact test. PERR, primary efficacy renal response.

**Table 3:** NLRP3 immunohistochemistry staining in baseline kidney biopsies from 20 patients with biopsy-proven active lupus nephritis.

NLRP3 IHC	PERR N = 13	No PERR N = 7	*p*-Value
Inflammatory cells			
Negative, n (%)	4 (30.8)	3 (42.8)	0.0426 ^‡^
1+, n (%)	7 (53.8)	0 (0.0)	
2+, n (%)	2 (15.4)	4 (57.2)	
Tubular cells			
Negative, n (%)	0	0	>0.999 ^‡^
1+, n (%)	3 (23.1)	2 (28.6)	
2+, n (%)	10 (76.9)	5 (71.4)	
Podocytes			
Negative, n (%)	2 (15.3)	1(14.3)	0.7676 ^‡^
1+, n (%)	10 (76.9)	4 (57.1)	
2+, n (%)	1 (7.8)	2 (28.6)	

^‡^ Fisher’s exact test. PERR, primary efficacy renal response. IHC, immunohistochemistry.

**Table 4:** Follow up of 20 patients with lupus nephritis after 6 months and 12 months of biopsy-proven active lupus nephritis and immunosuppressive treatment.

	PERR N = 13	No PERR N = 7	*p*-Value
**6 months**			
Serum Cr (mg/dL), mean ± SD	0.68 ± 0.12	0.94 ± 0.35	0.1046 *
eGFR (mL/min/1.73 m^2^),	114.6 ± 15.0	88.9 ± 29.1	0.0165 *
Proteinuria (g/24 h), mean ± SD	0.4 (0.2–1.0)	3.6 (0.9–4.1)	0.0037 ^¥^
Serum albumin (g/dL), mean ± SD	3.7 ± 0.4	3.1 ± 0.7	0.0218 *
C3 (mg/dL), mean ± SD	92.0 ± 18.7	86.3 ± 19.5	0.5917 *
C4 (mg/dL), median (range)	15.6 ± 6.2	18.7 ± 9.0	0.4863 *
SLEDAI, median (range)	2 (0–4)	4 (2–6)	0.1105 ^¥^
Use of ACEI/ARB, n (%)	12 (92.3)	6 (85.7)	>0.999 ^¥^
Use of hydroxychloroquine, n (%)	13 (100)	7 (100)	
Immunosuppressive treatment			
Corticosteroids dose (mg/kg/day)	0.3 ± 0.2	0.3 ± 0.2	0.8553 *
Cyclophosphamide, n (%)	1 (7.7)	2 (28.5)	0.2702 ^‡^
MMF, n (%)	12 (92.3)	5 (71.4)	
MMF dose (g/day), mean ± SD	2.0 (1.0–2.0)	1.5 (1.0–2.0)	0.5225 ^¥^
**12 months**			
Serum Cr (mg/dL), median (range)	0.71 ± 0.16	0.94 ± 0.43	0.2247 *
eGFR (mL/min/1.73 m^2^), mean ± SD	108.1 ± 16.2	91.4 + 34.4	0.1573 *
Proteinuria (g/24 h), mean ± SD	0.3 ± 0.2	2.4 ± 1.5	0.0001 *
Serum albumin (g/dL), mean ± SD	4.0 ± 0.25	3.4 ± 0.7	0.0096 *
C3 (mg/dL), mean ± SD	97.9 ± 11.9	91.4 ± 19.2	0.4648 *
C4 (mg/dL), median (range)	17.3 ± 6.7	19.3 ± 12.7	0.7172 *
SLEDAI, mean ± SD	0 (0–2)	6 (4–6)	0.0004 ^¥^
Use of ACEI/ARB, n (%)	11 (84.6)	7 (100)	0.5211 ^‡^
Use of hydroxychloroquine, n (%)	13 (100)	7 (100)	
Immunosuppressive treatment			
Corticosteroids dose (mg/kg/day)	0 (0.0–0.15)	0.1 (0.0–0.3)	0.2866 ^¥^
Cyclophosphamide, n (%)	0 (0)	0 (0)	
MMF, n (%)	13 (100)	7 (100)	
MMF dose (g/day), mean ± SD	1.0 (1.0–2.0)	2.0 (1.5–2.0)	0.2642 ^¥^

Cr, creatinine; eGFR, estimated glomerular filtration rate; SLEDAI, systemic lupus erythematosus disease activity index; ACEI, angiotensin-converting enzyme inhibitors; ARB, angiotensin receptor blocker; MMF, mycophenolate mofetil. eGFR was calculated using the Chronic Kidney Disease Epidemiology Collaboration (CKD-EPI) equation. Values are expressed as median with range (25th–75th percentile) or mean ± standard deviation (SD). * *t*-test; ^¥^ Mann–Whitney test; ^‡^ Fisher’s exact test.

## Data Availability

The original contributions presented in this study are included in the article. Further inquiries can be directed to the corresponding authors.

## References

[1] Hanly J.G., O’Keeffe A.G., Su L., Urowitz M.B., Romero-Diaz J., Gordon C., Bae S.-C., Bernatsky S., Clarke A.E., Wallace D.J. (2016). The Frequency and Outcome of Lupus Nephritis: Results from an International Inception Cohort Study. Rheumatology.

[2] Lim S.S., Bayakly A.R., Helmick C.G., Gordon C., Easley K.A., Drenkard C. (2014). The Incidence and Prevalence of Systemic Lupus Erythematosus, 2002–2004: The Georgia Lupus Registry. Arthritis Rheumatol..

[3] Bastian H.M., Roseman J.M., Mcgwin G., Alarcón G.S., Friedman A.W., Fessler B.J., Baethge B.A., Reveille J.D. (2002). Systemic Lupus Erythematosus in Three Ethnic Groups. XII. Risk Factors for Lupus Nephritis after Diagnosis. Lupus.

[4] Cervera R., Khamashta M.A., Font J., Sebastiani G.D., Gil A., Lavilla P., Mejía J.C., Aydintug A.O., Chwalinska-Sadowska H., de Ramón E. (2003). Morbidity and Mortality in Systemic Lupus Erythematosus During a 10-Year Period. Medicine.

[5] Gupta S., Kaplan M.J. (2021). Bite of the Wolf: Innate Immune Responses Propagate Autoimmunity in Lupus. J. Clin. Investig..

[6] Zhang H., Liu L., Li L. (2018). Lentivirus-Mediated Knockdown of FcγRI (CD64) Attenuated Lupus Nephritis via Inhibition of NF-ΚB Regulating NLRP3 Inflammasome Activation in MRL/Lpr Mice. J. Pharmacol. Sci..

[7] Ke P.F., Zhu Y.T., Cao S.L., Wang Y., Wu S.T., He Q.Q., Liang L.F., Li J.C. (2024). Identification of Pattern Recognition Receptor Genes in Peripheral Blood Mononuclear Cells and Monocytes as Biomarkers for the Diagnosis of Lupus Nephritis. Clin. Chim. Acta.

[8] Wang Z., Zhang S., Xiao Y., Zhang W., Wu S., Qin T., Yue Y., Qian W., Li L. (2020). NLRP3 Inflammasome and Inflammatory Diseases. Oxid. Med. Cell. Longev..

[9] Chen F.-f., Liu X.-t., Tao J., Mao Z.-m., Wang H., Tan Y., Qu Z., Yu F. (2023). Renal NLRP3 Inflammasome Activation Is Associated with Disease Activity in Lupus Nephritis. Clin. Immunol..

[10] Koka S., Xia M., Zhang C., Zhang Y., Li P.L., Boini K.M. (2019). Podocyte NLRP3 Inflammasome Activation and Formation by Adipokine Visfatin. Cell. Physiol. Biochem..

[11] Ummarino D. (2017). NLRP3 Inflammasome Ignites Podocyte Dysfunction. Nat. Rev. Rheumatol..

[12] Vilaysane A., Chun J., Seamone M.E., Wang W., Chin R., Hirota S., Li Y., Clark S.A., Tschopp J., Trpkov K. (2010). The NLRP3 Inflammasome Promotes Renal Inflammation and Contributes to CKD. J. Am. Soc. Nephrol..

[13] Wang W., Wang X., Chun J., Vilaysane A., Clark S., French G., Bracey N.A., Trpkov K., Bonni S., Duff H.J. (2013). Inflammasome-Independent NLRP3 Augments TGF-β Signaling in Kidney Epithelium. J. Immunol..

[14] Lamkanfi M., Dixit V.M. (2014). Mechanisms and Functions of Inflammasomes. Cell.

[15] Kelley N., Jeltema D., Duan Y., He Y. (2019). The NLRP3 Inflammasome: An Overview of Mechanisms of Activation and Regulation. Int. J. Mol. Sci..

[16] Martinon F., Burns K., Rg Tschopp J. (2002). The Inflammasome: A Molecular Platform Triggering Activation of Inflammatory Caspases and Processing of ProIL-That They Possess Several Distinct Protein/Protein Inter-Action Domains Which Are Used to Assemble Large Multi-Component Complexes. Apaf-1, for e. Mol. Cell.

[17] Liu X., Zhang Z., Ruan J., Pan Y., Magupalli V.G., Wu H., Lieberman J. (2016). Inflammasome-Activated Gasdermin D Causes Pyroptosis by Forming Membrane Pores. Nature.

[18] Ke Q., Greenawalt A.N., Manukonda V., Ji X., Tisch R.M. (2023). The Regulation of Self-Tolerance and the Role of Inflammasome Molecules. Front. Immunol..

[19] Pontillo A., Girardelli M., Kamada A.J., Pancotto J.A.T., Donadi E.A., Crovella S., Sandrin-Garcia P. (2012). Polimorphisms in Inflammasome Genes Are Involved in the Predisposition to Systemic Lupus Erythematosus. Autoimmunity.

[20] da Cruz H.L.A., Cavalcanti C.A.J., de Azêvedo Silva J., de Lima C.A.D., Fragoso T.S., Barbosa A.D., Dantas A.T., de Ataíde Mariz H., Duarte A.L.B.P., Pontillo A. (2020). Differential Expression of the Inflammasome Complex Genes in Systemic Lupus Erythematosus. Immunogenetics.

[21] Lee Y.H., Bae S.-C. (2016). Association between Functional NLRP3 Polymorphisms and Susceptibility to Autoimmune and Inflammatory Diseases: A Meta-Analysis. Lupus.

[22] Su Z., Niu Q., Huang Z., Yang B., Zhang J. (2020). Association of Nucleotide-Binding Oligomerization Domain-like Receptor Family Pyrin Domain-Containing Protein 3 Polymorphisms with Systemic Lupus Erythematosus Disease Activity and Biomarker Levels. Medicine.

[23] Tsai P.Y., Ka S.M., Chang J.M., Chen H.C., Shui H.A., Li C.Y., Hua K.F., Chang W.L., Huang J.J., Yang S.S. (2011). Epigallocatechin-3-Gallate Prevents Lupus Nephritis Development in Mice via Enhancing the Nrf2 Antioxidant Pathway and Inhibiting NLRP3 Inflammasome Activation. Free Radic. Biol. Med..

[24] Su B., Ye H., You X., Ni H., Chen X., Li L. (2018). Icariin Alleviates Murine Lupus Nephritis via Inhibiting NF-ΚB Activation Pathway and NLRP3 Inflammasome. Life Sci..

[25] Lin T.J., Wu C.Y., Tsai P.Y., Hsu W.H., Hua K.F., Chu C.L., Lee Y.C., Chen A., Lee S.L., Lin Y.J. (2019). Accelerated and Severe Lupus Nephritis Benefits from M1, an Active Metabolite of Ginsenoside, by Regulating NLRP3 Inflammasome and T Cell Functions in Mice. Front. Immunol..

[26] Yang S., Hsu W., Wu C., Shang H., Liu F., Chen A., Hua K., Ka S. (2020). Accelerated, Severe Lupus Nephritis Benefits from Treatment with Honokiol by Immunoregulation and Differentially Regulating NF-κB/NLRP3 Inflammasome and Sirtuin 1/Autophagy Axis. FASEB J..

[27] Zhao J., Wang H., Dai C., Wang H., Zhang H., Huang Y., Wang S., Gaskin F., Yang N., Man Fu S. (2013). P2X 7 Blockade Attenuates Murine Lupus Nephritis by Inhibiting Activation of the NLRP3/ASC/Caspase 1 Pathway. Arthritis Rheum..

[28] Bonomini F., Dos Santos M., Veronese F.V., Rezzani R. (2019). NLRP3 Inflammasome Modulation by Melatonin Supplementation in Chronic Pristane-Induced Lupus Nephritis. Int. J. Mol. Sci..

[29] Aringer M., Costenbader K., Daikh D., Brinks R., Mosca M., Ramsey-goldman R., Smolen J.S., Wofsy D., Boumpas D.T., Kamen D.L. (2019). 2019 European League Against Rheumatism/American College of Rheumatology Classification Criteria for Systemic Lupus Erythematosus. Arthritis Rheumatol..

[30] Gladman D.D., Ibañez D., Urowitz M.B. (2002). Systemic Lupus Erythematosus Disease Activity Index 2000. J. Rheumatol..

[31] Weening J.J., D’Agati V.D., Schwartz M.M., Seshan S.V., Alpers C.E., Appel G.B., Balow J.E., Bruijn J.A., Cook T., Ferrario F. (2004). The Classification of Glomerulonephritis in Systemic Lupus Erythematosus Revisited. J. Am. Soc. Nephrol..

[32] Weening J.J., D’agati V.D., Schwartz M.M., Seshan S.V., Alpers C.E., Appel G.B., Balow J.E., Bruijn J.A., Cook T., Ferrario F. (2004). The Classification of Glomerulonephritis in Systemic Lupus Erythematosus Revisited. Kidney Int..

[33] Bajema I.M., Wilhelmus S., Alpers C.E., Bruijn J.A., Colvin R.B., Cook H.T., D’Agati V.D., Ferrario F., Haas M., Jennette J.C. (2018). Revision of the International Society of Nephrology/Renal Pathology Society Classification for Lupus Nephritis: Clarification of Definitions, and Modified National Institutes of Health Activity and Chronicity Indices. Kidney Int..

[34] Mulay S.R. (2019). Multifactorial Functions of the Inflammasome Component NLRP3 in Pathogenesis of Chronic Kidney Diseases. Kidney Int..

[35] Lorenz G., Darisipudi M.N., Anders H.J. (2014). Canonical and Non-Canonical Effects of the NLRP3 Inflammasome in Kidney Inflammation and Fibrosis. Nephrol. Dial. Transplant..

[36] Guo C., Fu R., Zhou M., Wang S., Huang Y., Hu H., Zhao J., Gaskin F., Yang N., Fu S.M. (2019). Pathogenesis of Lupus Nephritis: RIP3 Dependent Necroptosis and NLRP3 Inflammasome Activation. J. Autoimmun..

[37] Takano Y., Yamauchi K., Hayakawa K., Hiramatsu N., Kasai A., Okamura M., Yokouchi M., Shitamura A., Yao J., Kitamura M. (2007). Transcriptional Suppression of Nephrin in Podocytes by Macrophages: Roles of Inflammatory Cytokines and Involvement of the PI3K/Akt Pathway. FEBS Lett..

[38] Fu R., Guo C., Wang S., Huang Y., Jin O., Hu H., Chen J., Xu B., Zhou M., Zhao J. (2017). Podocyte Activation of NLRP3 Inflammasomes Contributes to the Development of Proteinuria in Lupus Nephritis. Arthritis Rheumatol..

[39] Zhang C., Boini K.M., Xia M., Abais J.M., Li X., Liu Q., Li P.L. (2012). Activation of Nod-like Receptor Protein 3 Inflammasomes Turns on Podocyte Injury and Glomerular Sclerosis in Hyperhomocysteinemia. Hypertension.

[40] Vesey D.A., Cheung C.W.Y., Cuttle L., Endre Z.A., Gobé G., Johnson D.W. (2002). Interleukin-Lβ Induces Human Proximal Tubule Cell Injury, α-Smooth Muscle Actin Expression and Fibronectin Production. Kidney Int..

[41] Liu Y., Lei H., Zhang W., Xing Q., Liu R., Wu S., Liu Z., Yan Q., Li W., Liu X. (2023). Pyroptosis in Renal Inflammation and Fibrosis: Current Knowledge and Clinical Significance. Cell Death Dis..

[42] Huang G., Zhang Y., Zhang Y., Ma Y. (2023). Chronic Kidney Disease and NLRP3 Inflammasome: Pathogenesis, Development and Targeted Therapeutic Strategies. Biochem. Biophys. Rep..

[43] Choi S.E., Fogo A.B., Lim B.J. (2023). Histologic Evaluation of Activity and Chronicity of Lupus Nephritis and Its Clinical Significance. Kidney Res. Clin. Pract..

[44] Parodis I., Tamirou F., Houssiau F.A. (2020). Prediction of Prognosis and Renal Outcome in Lupus Nephritis. Lupus Sci. Med..

[45] Nakagawa S., Toyama T., Iwata Y., Oshima M., Ogura H., Sato K., Yamamura Y., Miyakawa T., Kitajima S., Hara A. (2021). The Relationship between the Modified National Institute of Health Activity and Chronicity Scoring System, and the Long-Term Prognosis for Lupus Nephritis: A Retrospective Single-Center Study. Lupus.

[46] Zhang W., Yuan M., Hong L., Zhou Q., Chen W., Yang S., Yang Q., Chen W., Yu X. (2016). Clinical Outcomes of Lupus Nephritis Patients with Different Proportions of Crescents. Lupus.

[47] Ludwig-Portugall I., Bartok E., Dhana E., Evers B.D.G., Primiano M.J., Hall J.P., Franklin B.S., Knolle P.A., Hornung V., Hartmann G. (2016). An NLRP3-Specific Inflammasome Inhibitor Attenuates Crystal-Induced Kidney Fibrosis in Mice. Kidney Int..

[48] Guo H., Bi X., Zhou P., Zhu S., Ding W. (2017). NLRP3 Deficiency Attenuates Renal Fibrosis and Ameliorates Mitochondrial Dysfunction in a Mouse Unilateral Ureteral Obstruction Model of Chronic Kidney Disease. Mediat. Inflamm..

[49] Wu X., Yang J., Wu J., Yang X. (2024). Therapeutic Potential of MCC950, a Specific Inhibitor of NLRP3 Inflammasome in Systemic Lupus Erythematosus. Biomed. Pharmacother..

[50] Fu R., Xia Y., Li M., Mao R., Guo C., Zhou M., Tan H., Liu M., Wang S., Yang N. (2019). Pim-1 as a Therapeutic Target in Lupus Nephritis. Arthritis Rheumatol..

[51] Wu C.-Y., Hua K.-F., Chu C.-L., Yang S.-R., Arbiser J.L., Yang S.-S., Lin Y.-C., Liu F.-C., Yang S.-M., Ka S.-M. (2020). Tris DBA Ameliorates Accelerated and Severe Lupus Nephritis in Mice by Activating Regulatory T Cells and Autophagy and Inhibiting the NLRP3 Inflammasome. J. Immunol..

[52] Zhao J., Zhang H., Huang Y., Wang H., Wang S., Zhao C., Liang Y., Yang N. (2013). Bay11-7082 Attenuates Murine Lupus Nephritis via Inhibiting NLRP3 Inflammasome and NF-ΚB Activation. Int. Immunopharmacol..

[53] Karakaya T., Slaufova M., Di Filippo M., Hennig P., Kündig T., Beer H.-D. (2024). CARD8: A Novel Inflammasome Sensor with Well-Known Anti-Inflammatory and Anti-Apoptotic Activity. Cells.

[54] Razmara M., Srinivasula S.M., Wang L., Poyet J.L., Geddes B.J., Distefano P.S., Bertin J., Alnemri E.S. (2002). CARD-8 Protein, a New CARD Family Member That Regulates Caspase-1 Activation and Apoptosis. J. Biol. Chem..

[55] Ito S., Hara Y., Kubota T. (2014). CARD8 Is a Negative Regulator for NLRP3 Inflammasome, but Mutant NLRP3 in Cryopyrin-Associated Periodic Syndromes Escapes the Restriction. Arthritis Res. Ther..

[56] Mao L., Kitani A., Similuk M., Oler A.J., Albenberg L., Kelsen J., Aktay A., Quezado M., Yao M., Montgomery-Recht K. (2018). Loss-of-Function CARD8 Mutation Causes NLRP3 Inflammasome Activation and Crohn’s Disease. J. Clin. Investig..

[57] Linder A., Bauernfried S., Cheng Y., Albanese M., Jung C., Keppler O.T., Hornung V. (2020). CARD8 Inflammasome Activation Triggers Pyroptosis in Human T Cells. EMBO J..

[58] Taabazuing C.Y., Griswold A.R., Bachovchin D.A. (2020). The NLRP1 and CARD8 Inflammasomes. Immunol. Rev..

[59] Inker L.A., Eneanya N.D., Coresh J., Tighiouart H., Wang D., Sang Y., Crews D.C., Doria A., Estrella M.M., Froissart M. (2021). New Creatinine- and Cystatin C–Based Equations to Estimate GFR without Race. N. Engl. J. Med..

[60] Rovin B.H., Ayoub I.M., Chan T.M., Liu Z.-H., Mejía-Vilet J.M., Floege J. (2024). KDIGO 2024 Clinical Practice Guideline for the Management of LUPUS NEPHRITIS. Kidney Int..

[61] Tamirou F., Lauwerys B.R., Dall’Era M., Mackay M., Rovin B., Cervera R., Houssiau F.A. (2015). A Proteinuria Cut-off Level of 0.7 g/Day after 12 Months of Treatment Best Predicts Long-Term Renal Outcome in Lupus Nephritis: Data from the MAINTAIN Nephritis Trial. Lupus Sci. Med..

[62] Dall’Era M., Cisternas M.G., Smilek D.E., Straub L., Houssiau F.A., Cervera R., Rovin B.H., MacKay M. (2015). Predictors of Long-Term Renal Outcome in Lupus Nephritis Trials: Lessons Learned from the Euro-Lupus Nephritis Cohort. Arthritis Rheumatol..

[63] Furie R., Rovin B.H., Houssiau F., Malvar A., Teng Y.K.O., Contreras G., Amoura Z., Yu X., Mok C.-C., Santiago M.B. (2020). Two-Year, Randomized, Controlled Trial of Belimumab in Lupus Nephritis. N. Engl. J. Med..

[64] Manchester K.L. (1996). Use of UV Methods for Measurement of Protein and Nucleic Acid Concentrations. Biotechniques.

[65] Schmittgen T.D., Livak K.J. (2008). Analyzing Real-Time PCR Data by the Comparative CT Method. Nat. Protoc..

